# Particle Swarm Algorithm-Based Analysis of Pelvic Dynamic MRI Images in Female Stress Urinary Incontinence

**DOI:** 10.1155/2021/8233511

**Published:** 2021-07-30

**Authors:** Dongfang Su, Yufang Wen, Qing Lin

**Affiliations:** Department of Obstetrics and Gynecology, People's Hospital of Xinzhou District, Wuhan 430400, Hubei, China

## Abstract

This work aimed to study the application of pelvic floor dynamic images of magnetic resonance imaging (MRI) based on the particle swarm optimization (PSO) algorithm in female stress urinary incontinence (SUI). 20 SUI female patients were selected as experimental group, and another 20 healthy females were taken as controls. PSO algorithm, K-nearest neighbor (KNN) algorithm, and back propagation neural network (BPNN) algorithm were adopted to construct the evaluation models for comparative analysis, which were then applied to 40 cases of female pelvic floor dynamic MRI images. It was found that the model proposed had relatively high prediction accuracy in both the training set (87.67%) and the test set (88.46%). In contrast to the control group, there were considerable differences in abnormal urethral displacement, urethral length changes, bladder prolapse, and uterine prolapse in experimental patients (*P* < 0.05). After surgery, the change of urethral inclination angle was evidently reduced (*P* < 0.05). To sum up, MRI images can be adopted to assess the occurrence of female SUI with abnormal urethral displacement, shortening of urethra length, bladder prolapse, and uterine prolapse. After surgery, the abnormal urethral movement was slightly improved, but there was no obvious impact on bladder prolapse and uterine prolapse.

## 1. Introduction

SUI refers to the involuntary leakage of urine from the external urethra when the abdominal pressure increases after sneezing or coughing [1]. With the increase of age, the prevalence of SUI patients also increases, the high incidence age is concentrated in the age of 45 to 55 years, and the number of births is positively correlated with the occurrence of urinary incontinence [2]. From a clinical point of view, the pelvic floor covers a wide range, including all muscle fascia layers from the pelvic peritoneum to the perineum skin. From top to bottom, there are peritoneum, pelvic fascia, pelvic diaphragm, urogenital septum, external anal sphincter, and superficial urogenital muscles [3]. The striated muscles that make up the pelvic floor are the levator ani muscle, puborectalis muscle, coccyx muscle, anterior perineum, and posterior perineum. The pelvic floor has dual functions, namely, supporting the pelvic and abdominal organs and coordinating defecation [4].

With the rapid development of computer and imaging technology, MRI, as another clinical medical imaging technology after radiology relay ultrasound and X-ray plain film, its development, and application has developed gradually [5]. Dynamic contrast-enhanced MRI technology has been maturely applied to female stress urinary incontinence [6]. PSO algorithm is a search algorithm with fast convergence speed and high accuracy used in recent years [7]. In the early stage of the search, to improve the global search capability of the particle swarm and enable the particle swarm to find the optimal solution range area as quickly as possible, the inertia weight is set to a relatively large value. In the later stage of the search, to control the speed of the particle swarm, the inertia weight value is reduced, so that the particle swarm can be accurately searched locally [8].

In this work, pelvic floor dynamic MRI images based on the PSO algorithm were acquired, and evaluation models were built via KNN and BPNN for comparative analysis, which were then applied to the image diagnosis of 20 cases of SUI patients. The application effect of MRI images based on PSO algorithm in SUI patients was comprehensively evaluated after images of different groups of patients were compared.

## 2. Materials and Methods

### 2.1. Selection of Research Subjects

20 pathologically confirmed SUI patients who were admitted to the hospital from February 15, 2019, to April 20, 2020, were taken as the experimental patients. Another 20 healthy women were selected as the control group, and the age range was 25–68 years old. There was no significant difference in general data between the two groups, which were comparable. The study had been approved by the Medical Ethics Committee of Hospital, and the patients and their families understood the situation of the study and signed the informed consent forms.

#### 2.1.1. Inclusion Criteria

I, patients have not received pelvic surgery; II, patients without contraindications to MRI scanning; III, patients without pelvic placeholder; IV, patients without urinary system disease; V, patients with clear consciousness and could be checked normally.

#### 2.1.2. Exclusion Criteria

I, patients with mental illness; II, patients with incomplete clinical data; III, patients who withdrew from the experiment due to their own reasons.

### 2.2. Dynamic-Enhanced Magnetic Resonance Scan

The MR Prisma 3.0 magnetic resonance machine produced by Siemens in Germany was utilized to examine the patients. The procedure was explained to the patient ahead of the scan, and the patient kept breathing calmly. During the examination, the patient was placed in the supine position, and axial and sagittal MRI images of the pelvic cavity were obtained. A high-pressure syringe was adopted to inject 0.2 mmol/L contrast enhancer Gd-DTPA (Bayer Healthcare, Berlin, Germany) into the dorsal hand vein at an injection rate of 2.5 mL/s, and then the same amount of normal saline was injected. Eight time-phases were collected consecutively, and the scanning time was 60 s. Scanning parameters: the matrix was 251 × 251, the layer thickness was 3.5 mm, the field of vision was 25 × 25 cm, the flip angle was 15°, and the layer spacing was 6.1 mm.

### 2.3. Observation Sites

The evaluation of pelvic floor function mainly observed the pubococcygeal line (PCL). The pubococcygeal line is the line from the lower margin of the symphysis pubis to the last two coccyges. Sagittal position was adopted to determine the H-line, M-line, length of urethra, and posterior pubic space. Changes in angle were measured for the angle of the levator plate, the angle of urethra inclination, the posterior angle of urethra and bladder, and the angle of the connection between the bladder and urethra and PCL. Statistical data were then obtained.

The signal strength of the urethra supporting ligament structure in the resting and exerting state of female patients was observed, and the controls were compared with experimental patients in the resting and exerting state. Before and after the surgery, SUI patients took sagittal positions in resting and exerting states, respectively, and the H-line, M-line, urethra length, and posterior pubic space were measured. Changes in angle were measured for the angle of the levator plate, the angle of urethra inclination, the posterior angle of urethra and bladder, and the angle of the connection between the bladder and urethra and PCL. Statistical data were then obtained.

### 2.4. Evaluation Criteria for Changes in Observation Parts

Urethral assessment criteria: the normal urethral length is located above the pubic symphysis, and the length is about 66% of the total length according to standard of the resting state and the forced state of the sagittal MRI. For the displacement angle of the urethra, the normal displacement angle is less than 30°. For the angle of bladder movement, the angle between the back of the bladder and the urethra was observed. Diagnostic criteria for bladder prolapse and uterine prolapse: the changes in the angle between the bladder and PCL under the state of exertion were detected by sagittal MRI, and the same for uterine prolapse. The bottom of the basin was loose, and H-line and M-line were observed. H-line refers to the connecting line between the pubic symphysis and the anorectum, which can reflect the size of the genital hole. The M-line refers to the vertical distance between the anorectum and the PCL line, which can reflect the changes in the levator ani muscle.

### 2.5. PSO Algorithm

PSO algorithm refers to that, in a one-dimensional optimized vector space, it is assumed that there are particles forming a particle group for target search in the *D*-dimensional search space. Each particle has a corresponding position. PSO algorithm needs to use the fitness function to judge the iteration quality of the particle. According to the mathematical expression of the objective function, the fitness value of the particle can be calculated. By calculating the size of the particle fitness value, the pros and cons are judged. The flight speed and position update of particles are calculated by the following equation:

(1) $$\begin{array}{c} u_{i}^{d}=u_{i}^{d}+a_{1}S_{1}^{d}\left(q{\text{Good}}_{i}^{d}-x_{i}^{d}\right)+a_{2}S_{2}^{d}\left(h{\text{Good}}^{d}-x_{i}^{d}\right), \end{array}$$

(2) $$\begin{array}{c} x_{i}^{d}=x_{i}^{d}+u_{i}^{d}. \end{array}$$

In equations (1) and (2), *d* *=* 1, 2,…, *D*, *a*_1_ and *a*_2_ are the acceleration coefficients, which are uniformly distributed in the range of [0, 1]. *q*Good_*i*_^*d*^ represents the global optimal position of a single particle *i*, and *h*Good^*d*^ represents the global optimal position of the population. According to equations (1) and (2), the global search ability and local position search ability of particles are strengthened. Some scholars introduce inertial weight *v* and update equation (1) to obtain the following equation.

(3) $$\begin{array}{c} u_{i}^{d}=vu_{i}^{d}+a_{1}S_{1}^{d}\left(q{\text{Good}}_{i}^{d}-x_{i}^{d}\right)+a_{2}S_{2}^{d}\left(h{\text{Good}}^{d}-x_{i}^{d}\right). \end{array}$$

If the inertia weight in equation (3) is set to 0.6, the following equation can be obtained:

(4) $$\begin{array}{c} v=v_{\max}-\left(v_{\max}-v_{\min}\right)\frac{h}{H}. \end{array}$$

In equation (4), *v*_max_ represents the maximum inertia weight, which is usually set to 0.8, *v*_min_ represents the minimum inertia weight, which is usually set to 0.6, *h* represents evolution algebra, and *H* represents the maximum value of evolution algebra. To improve the convergence of the PSO algorithm, some studies have introduced a shrinkage factor *χ*, and the update equation is changed to the following equations:

(5) $$\begin{array}{c} u_{i}^{d}=\chi \left[u_{i}^{d}{+}_{1}S_{1}^{d}\left(q{\text{Good}}_{i}^{d}-x_{i}^{d}\right)+a_{2}S_{2}^{d}\left(h{\text{Good}}^{d}-x_{i}^{d}\right)\right], \end{array}$$

(6) $$\begin{array}{c} \chi =\frac{2}{\left|2-\zeta -\sqrt{\zeta^{2}-4\zeta }\right|}. \end{array}$$

In equations (5) and (6), *ζ*=*a*_1_+*a*_2_ > 4.0. Usually, the shrinkage factor is set to *χ* = 0.729. The shrinkage factor is essentially the same as the inertia weight. The fuzzy adaptive inertia weight is introduced into the PSO algorithm, and equation (7) is obtained.

(7) $$\begin{array}{c} v=0.5+S*0.5. \end{array}$$

In equation (7), *v* represents the inertia weight and *S* is uniformly distributed in the range of [0, 1]. The algorithm is improved with the topology structure, and the topology of the particle *i*'s location is updated. The following equation is obtained:

(8) $$\begin{array}{c} u_{i}^{d}=vu_{i}^{d}+a_{1}S_{1}^{d}\left(q{\text{Good}}_{i}^{d}-x_{i}^{d}\right)+a_{2}S_{2}^{d}\left(M{\text{Good}}^{d}-x_{i}^{d}\right). \end{array}$$

In equation (8), *M*Good^*d*^ represents the global historical optimal position of the position topology of the particle *i*.

### 2.6. Statistical Methods

Data were processed by SPSS20.0 version statistical software analysis, mean ± standard deviation ($\bar{x}$ ± *s*) was how measurement data were expressed, and percentage (%) was how count data were expressed. PSO algorithm was compared with the other two algorithms regarding the parameters, and the related angle changes of experimental group and controls were compared. The intragroup dynamic-static comparative study of each observation index was tested by paired *t*-test. The difference was considerable at *P* < 0.05.

## 3. Results

### 3.1. Analysis of Empirical Results of PSO Algorithm

**Figure 1:**
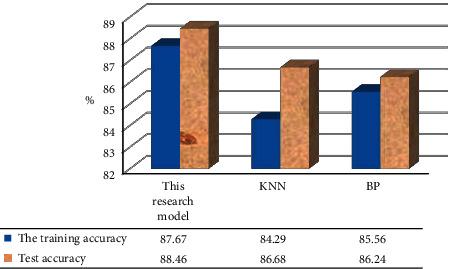
Contrast of training accuracy and test accuracy of three algorithms.

To prove the superiority of the algorithm proposed in this study, the evaluation models constructed by the KNN algorithm and BPNN algorithm were compared and analyzed in Figure 1. The model proposed had relatively high prediction accuracy in both the training set (87.67%) and the test set (88.46%), indicating that the PSO algorithm can accurately improve the accuracy of training samples and test samples. Since the data in the test would be diversified with the changes of many factors, the model proposed in this research was proved to have good practicability.

### 3.2. MRI Manifestations of SUI Patients

**Figure 2:**
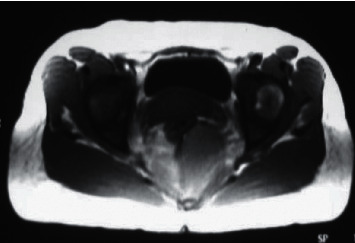
Pubourethral ligament. Note: the pubic urethral ligament was loose, without tightness.

**Figure 3:**
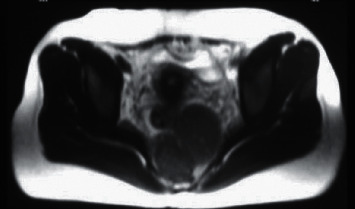
Bladder prolapse and uterine prolapse. Note: abnormal pubic bone displacement, bladder and uterine prolapse.

In females of the control group, the urethral support structure exhibited lower signals at both T1WI and T2WI. Three groups of ligament structures can be clearly identified, namely, the paraurethral ligament, the periurethral ligament, and the pubourethral ligament. The paraurethral ligament showed low signal, one end was connected to the urethral wall, and the other end was connected to the periurethral ligament. For people with paraurethral ligaments, it can only detect one side, and for some people, it can detect both sides. The periurethral ligament showed fan-shaped low signal, which was clearly visible on the ventral side of the urethra, connected to both sides of the puborectalis muscle, and was seen in the middle of the urethra. The pubic urethral ligament was short-line low signal, connected to the periurethral ligament, and distributed asymmetrically, leaning to one side. MRI was adopted to observe in sagittal position, and it was found that the pubic urethral ligament was divided into proximal, middle, and distal ends. The support structure of the urethra of SUI patients would undergo significant changes. The typical manifestation was ligament laxity and abnormalities in multiple groups of ligaments. In this study, the ligaments around the urethra were loosened and showed irregular waves. Part of them was interrupted and no longer continuous, which was separated from the urethral wall. The pubis and urethral ligament appeared loose, and there was no tightness (Figure 2). The overall structure of the pelvic floor was loose, leading to enlargement of the genital holes and loosening of the pelvic floor muscles, which increased the probability of organ prolapse (Figure 3).

### 3.3. Contrast of MRI Results of Two Groups of Females

**Figure 4:**
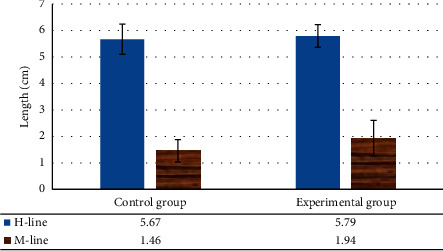
Contrast of H-line and M-line length between two groups of females.

**Figure 5:**
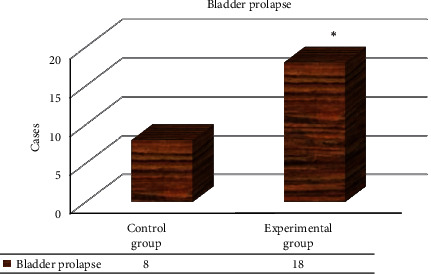
Contrast of bladder prolapse between two groups of females. Note: ^*∗*^*P* < 0.05 versus controls.

The H-line and M-line of the two groups of females were compared, and the difference was not substantial (*P* > 0.05) (Figure 4). However, in the state of exertion, in contrast to controls, the length of the H-line in experimental patients increased, and the value of the M-line also increased. The two groups of females were compared regarding the bladder prolapse, and the number of cases of bladder prolapse in experimental patients was evidently higher relative to controls, with considerable difference (*P* < 0.05) (Figure 5). The probability of uterine prolapse in SUI patients was also higher than that of controls, and the difference was substantial (*P* < 0.05).

### 3.4. Contrast of Related Changes between the Two Groups of Females

**Figure 6:**
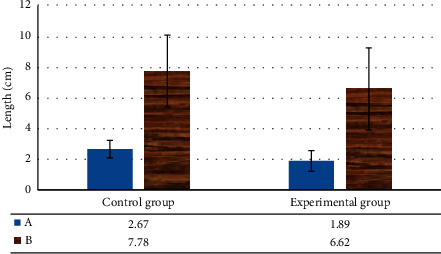
Length of resting urethra and posterior pubic space in SUI patients before and after surgery. A: resting urethral length; B: posterior pubic space.

**Figure 7:**
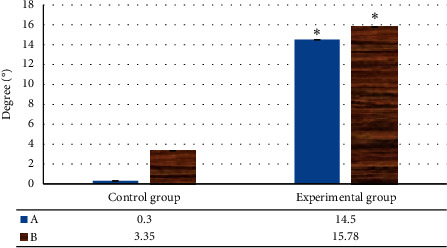
The changes of the levator plate angle and the distance from the bladder-urethral junction to the PCL of the two groups of females. A: change value of the levator plate angle; B: change value of distance from bladder-urethral junction to PCL. Note: ^*∗*^*P* < 0.05 versus controls.

**Figure 8:**
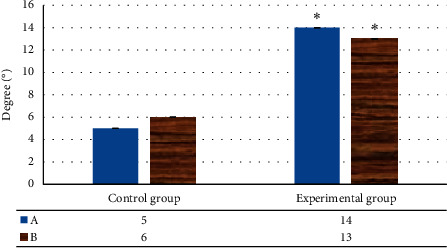
Changes in the posterior angle of the urethra and the urethra inclination angle of the two groups of females. A: change value of posterior urethral bladder angle; B: change value of urethral tilt angle. Note: ^*∗*^*P* < 0.05 versus controls.

The degree of change caused by urethral movement was the most common imaging feature in SUI patients. The urethra of experimental patients would move excessively from one side of the urethra to below the joint with the pubic bone in the state of exertion. It would cause the length of the urethra above the pubic symphysis to shorten, and the difference was highly notable relative the controls (*P* < 0.05) (Figure 6). The levator plate angle changes and the distance from the bladder-urethral junction to the PCL increased remarkably in experimental patients relative to controls, as shown in Figure 7. The changes in the posterior urethral bladder angle and the inclination angle of the urethra in experimental patients were greatly superior to controls, and the difference was remarkable (*P* < 0.05) (Figure 8).

### 3.5. Contrast of Conditions of SUI Patients before and after Surgery

**Figure 9:**
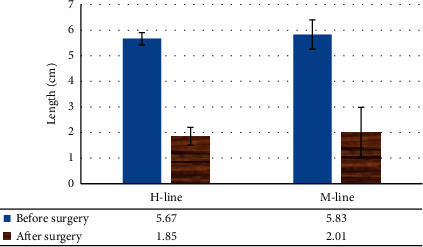
Contrast of H-line and M-line lengths of SUI patients before and after surgery.

**Figure 10:**
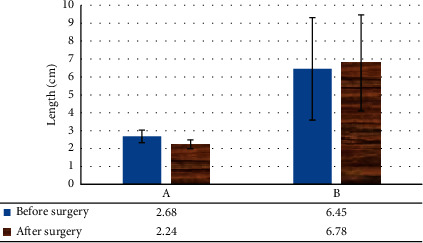
Changes in resting urethral length and retropubic space in patients with SUI before and after surgery. A: changes in the length of the resting urethra; B: changes in the posterior pubic space.

The females with SUI were compared before and after surgery in terms of the H-line length, M-line length, resting urethral length, and retropubic space, and no obvious difference was found (*P* > 0.05), as shown in Figures 9 and 10.

### 3.6. Correlative Angle Comparison of SUI Patients before and after Surgery

**Figure 11:**
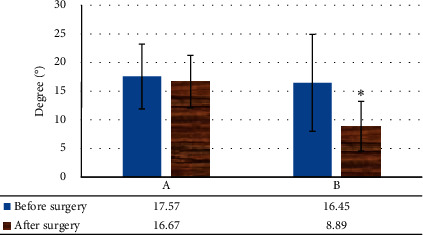
Changes in the angle of the levator plate and the bladder-urethral junction with the PCL in SUI patients before and after surgery. A: change value of levator plate angle; B: change value of angle between bladder urethra junction and PCL. Note: ^*∗*^*P* < 0.05 versus that before surgery.

**Figure 12:**
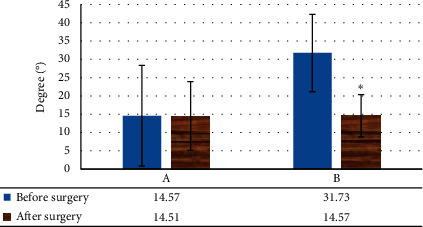
Changes of posterior urethral bladder angle and urethral inclination angle before and after surgery in SUI patients. A: change of posterior angle of urethra and bladder; B: change of urethral inclination angle. Note: ^*∗*^*P* < 0.05 versus that before surgery.

The levator plate angle did not change considerably, and the difference was not notable (*P* > 0.05). The change value of the angle between the bladder-urethral junction and the PCL was greatly reduced (*P* < 0.05) (Figure 11). No obvious change was found in the posterior angle of the urethra and bladder before and after the surgery, and the change in the inclination angle of the urethra was evidently reduced (*P* < 0.05) (Figure 12).

## 4. Discussion

The cause of SUI patients was that the external bladder and other factors worked together to reduce the maximum closure pressure of the urethra [9]. Niu et al. [10] introduced the topology structure into the PSO algorithm, which gradually developed from the initial static topology structure to the dynamic topology structure, thus solving the problem that even when the problem was complicated, and good optimization was achieved. The PSO algorithm was proposed, optimized, and compared with the KNN algorithm and the BPNN algorithm. The model proposed had a higher prediction accuracy than the other two algorithms, which was then applied to the pelvic floor dynamic MRI images of 20 females. The conventional sagittal scanning was conducted, and it was found that the whole process of the bottom of the pelvis could be clearly displayed, which confirmed the accuracy of the above research. The results of T1WI and T2WI images were also compared, and it was found that the overall anatomical structure of the urethra, vagina, and rectum can be clearly displayed. This work expanded the findings of other studies mentioned above.

In recent years, there have been various theoretical theories about the pathogenic mechanism of SUI. Radzimińska et al. [11] proposed the hammock hypothesis, which pointed out that the shape of a woman's urethra, bladder fascia, and vaginal anterior wall was similar to a “hammock.” When a healthy woman was under exertion, the nearby fascia and fascia tendon would contract, stretch the hammock, and the urethra would be stressed, which would increase the pressure in the abdomen and cause urine to be discharged from the body. Damage to the hammock structure of SUI patients would cause the urethra to fail to close normally, resulting in uncontrolled abnormal leakage of urine and urinary incontinence [12]. In anatomy, the internal structure of the pelvic floor was complicated. Watanabe et al. [13] proposed the theory of pelvic floor anatomy, which suggested that the pelvic floor was mainly divided into three parts, including pelvic fascia, pelvic floor ligaments, and pelvic floor muscles. The three major parts interacted to maintain the normal physiological functions of the pelvis. Abnormal urethral movement was the most common MRI imaging feature of SUI patients [14]. The length of the urethra played a very important role in controlling urine output. When SUI patients were resting in a supine position, about 50% of the urethra was located below the pubic symphysis. Compared with controls, the length of the urethra in experimental patients was slightly shortened. In the state of exertion, the urethra of SUI patients was almost completely located below the pubic symphysis.

Studies have reported that the incidence of pelvic bladder and uterine prolapse in SUI patients accounted for about 25% [15]. After surgery, pelvic bladder and uterine prolapse accounted for 15% [16]. In this study, pelvic floor dynamic MRI images showed that the incidence of pelvic bladder and uterine prolapse in experimental patients with SUI was greatly superior to controls (*P* < 0.05). In the state of exertion, the length of the H-line in SUI patients increased more in the experimental group than controls, indicating that the pelvic floor had begun to appear loose and the genital hole of the urinary system was enlarged. The increase in the value of the M-line indicated that the pelvic floor muscles were slack, which increased the probability of bladder and uterine prolapse. In the state of exertion, the dynamic changes of the related angle of the urethra after the surgery would have an impact on urinary control [17]. Some scholars have suggested that patients with SUI did not need to adjust the abnormal movement of the urethra during surgery [18]. After the operation, the abnormal movement angle of the urethra decreased immediately, and during the recovery period, the abnormal angle of the urethra gradually returned to its normal position [19]. The results of this work were consistent with the above studies. The levator plate angle, posterior urethral bladder angle, and urethral inclination angle of SUI patients in this study decreased after the surgery, and only the urethral inclination angle decreased significantly (*P* < 0.05).

## 5. Conclusion

Based on the PSO algorithm, the topology structure was introduced, and KNN and BPNN algorithms were adopted for comparisons. The results revealed that the prediction accuracy of the proposed model was high and had ideal practicability. The proposed model was applied to 40 cases of female pelvic floor dynamic MRI images, which could clearly show the whole pelvic floor. SUI patients would experience abnormal urethral displacement, shortened urethra length, bladder prolapse, and uterine prolapse. After the surgery, the abnormal movement of the urethra was slightly improved, but it had no major effect on the prolapse of the bladder and the uterus. The limitation of this work lies in the random grouping, and the influence of the severity of SUI on the urethra of patients is not taken into account, which may lead to some errors in the experimental results. In the later stage, patients' conditions are considered to be graded, so as to further study the correlation between SUI and pelvic organ prolapse. In conclusion, the use of pelvic floor dynamic MRI images can accurately assess the functional status of the entire pelvic floor of SUI patients and provide a good treatment for clinical diagnosis of SUI.

## Data Availability

The data used to support the findings of this study are available from the corresponding author upon request.
